# Socioeconomic disparities and cardio-cerebrovascular diseases: A nationwide cross-sectional study

**DOI:** 10.7189/jogh.14.04210

**Published:** 2024-10-11

**Authors:** Ji Woong Roh, SungA Bae, Moon-Hyun Kim, Je-Wook Park, Seok-Jae Heo, Minkwan Kim, Oh-Hyun Lee, Yongcheol Kim, Eui Im, Jae-Sun Uhm, In Hyun Jung, Deok-Kyu Cho, Donghoon Choi

**Affiliations:** 1 Department of Cardiology, Yongin Severance Hospital, Yonsei University College of Medicine, Yongin, Republic of Korea; 2 Division of Biostatistics, Department of Biomedical Systems Informatics, Yonsei University College of Medicine, Seoul, Republic of Korea; 3 Department of Cardiology, Severance Hospital, Yonsei University College of Medicine, Seodaemun-gu, Seoul, Republic of Korea

## Abstract

**Background:**

Although socioeconomic status (SES) is considered a risk factor for cardio-cerebrovascular diseases (CCVDs), few studies have examined this association. In this cross-sectional study, we aimed to assess the prevalence and trends of CCVDs across different SES groups over a 12-year period in a representative Korean population.

**Methods:**

We analysed 47 745 economically active adults aged ≥30 and <65 years from 97 622 patients in the Korean National Health and Nutrition Examination Survey (2007–18), where a new independent sample of the population was examined each year. We categorised the participants into four groups based on education level and income. The prevalence of hypertension, diabetes mellitus, dyslipidaemia, and CCVD, including angina, myocardial infarction, and stroke, was analysed at four-year intervals.

**Results:**

Average age, urban residence, white-collar occupation, and body mass index >30 increased, whereas CCVD prevalence did not change significantly (*P* = 0.410) over the study period. Low education (odds ratio (OR) = 1.24; 95% confidence interval (CI) = 1.04–1.47, *P* < 0.001) and low income (OR = 1.14; 95% CI = 1.02–1.28, *P* = 0.017) were significant determinants of CCVD in addition to existing traditional risk factors. CCVD prevalence was significantly higher in both the low-education and low-income groups compared to the high-education and high-income groups every four years, with no significant change in this gap over the study period (*P* = 0.239).

**Conclusions:**

Despite the increase in the elderly population and the prevalence of obesity, the incidence of CCVDs in Korea has remained unchanged. Individuals with low education or low income had a significantly higher prevalence of CCVD, with the lowest SES group, defined by both low education and low income, consistently having the highest prevalence of CCVDs.

Cardio-cerebrovascular diseases (CCVDs), including ischaemic heart disease and stroke, are the most common cause of death worldwide, according to the World Health Organization [1]. This trend continues to persist today [2]. Traditionally, hypertension, diabetes mellitus, dyslipidaemia, obesity, smoking, alcohol consumption, and lack of physical activity have been recognised as risk factors for CCVD [3]. Relatedly, there has been growing interest in socioeconomic status (SES), which has an inverse association with CCVD in high-income countries and is considered an important determinant of health outcomes [4]. Although SES is difficult to define, it can be broadly described as a composite of income, education, occupation, race, and social status [5]. Low SES is usually related to higher CCVD prevalence and lower survival rates owing to poor quality of care and limited access to the health care system [6].

As Korea entered the ranks of high-income countries, it experienced rapid development, with personal income tripling over the past 20 years. Conversely, the gap in SES has widened [7]. Developed countries have a predominantly private health care system, suggesting that the ownership of health insurance is an indicator of SES [8,9]. However, in Korea, the government regulates health care costs, and all citizens are required to enrol in the national health insurance system, while private insurance is purchased voluntarily. Therefore, despite the widening gap, Korean citizens of low SES have access to better health care than in any other high-income country [10]. Low SES is a known health determinant in high-income countries, but studies on this topic in high-income countries with different health insurance systems are still scarce. Therefore, we investigated the prevalence and associations of CCVD with major risk factors every four years from 2007 to 2018 after classifying SES into four groups according to educational and income levels, using the latest Korean National Health and Nutrition Examination Survey (KNHANES) data.

## METHODS

### Study protocol and recruitment

We analysed data from the KNHANES (2007–18), a national survey that assesses general health and nutritional status. The detailed design of the survey and its data collection methods have been previously reported [11]. Overall, 97 622 people participated in KNHANES IV (2007–09) through VII (2015–18). Considering the applicability of self-income, we excluded ages <30 (n = 31 608) and ≥65 (n = 18 279), since they were mainly economically inactive (Figure S1 in the **Online Supplementary Document**). Each survey wave had a unique sample of participants, meaning that different individuals were included in each survey period. This study protocol was approved by the Institutional Review Board of Yongin Severance Hospital (approval number: 2022-0455-001).

### Health interview and examination

KNHANES consists of three components: health interview, health examination, and nutritional survey. The health interview included detailed information on SES, covering age, sex, residence, job, education, household income, smoking status, alcohol consumption, and health care utilisation. A well-trained nurse conducted a health examination. According to standard protocols, systolic and diastolic blood pressure was measured three times every five minutes using a mercury sphygmomanometer (Baumanometer; Baum, New York, USA). Blood samples were collected after eight hours of fasting and processed according to the KNHANES protocols [11]. The blood was centrifuged at approx. 2500 to 3000 rpm for 15 minutes after separation with an 8 mL serum separation tube and maintained at room temperature for 30 minutes to estimate the lipid profile. Subsequently, 2 mL sodium fluoride tubes were mixed in a roller mixer for 10 minutes to analyse glucose levels. All samples were refrigerated (approx. 2–8°C), and glucose and cholesterol levels were measured by Advia 1650 and a Hitachi automatic analyser. In 2005, a quality control programme was implemented to monitor laboratory performance to ensure that values meet acceptable standards of precision and accuracy in a central laboratory. Since the programme began in 2005, we have been able to collect and analyse glucose and cholesterol levels for all patients.

We considered educational and income levels as SES indicators in our study. The former was categorised as high school graduation or lower. For the latter, we used the lower and upper household income cutoffs to divide participants into above and below-income categories, based on guidelines from the KNHANES provided by the Korea Disease Control and Prevention Agency. Specifically, we used the monthly income quartiles of the sample population by year to categorise participants (Table S1 in the **Online Supplementary Document**). The participants were divided into four groups according to educational and income levels. The reference group (group A) included participants with high education and high income, group B included participants with high education and low income, group C included participants with low education and high income, and group D represented participants with low education and low income.

### Definition of CCVD and risk factors

CCVD refers to a combination of angina, myocardial infarction (MI), and ischaemic or haemorrhagic stroke. The presence of diseases was investigated through interviews conducted at mobile examination centres. The information on the presence of each disease, the diagnosis by a physician, the timing of the initial diagnosis, and the current treatment status were all based on patient self-reports. Diagnoses were rigorously confirmed by whether a physician's diagnosis was reported, increasing the reliability of the data. Hypertension was identified as systolic or diastolic average blood pressure readings of ≥140/90 mm Hg or as the individual using anti-hypertensive medication. Diabetes mellitus criteria included fasting plasma glucose ≥125 mg/dL, HbA1c ≥6.5%, a prior diagnosis, or the use of anti-hyperglycaemic drugs or insulin. Dyslipidaemia was defined by total cholesterol ≥240 mg/dL, low-density lipoprotein cholesterol ≥160 mg/dL, or the use of cholesterol-lowering medications. We defined regular aerobic exercise as 30 minutes or more of physical activity five or more times per week. Obesity was determined as a body mass index (BMI)≥30 kg/m^2^, calculated as weight in kilograms divided by height in meters squared.

### Definitions of other socioeconomic indicators in KNHANES

In the KNHANES data, ‘urban’ refers to a city with a population of 50 000 or more, organised into administrative districts called ‘*dong*’. ‘Rural’ refers to a city or town with a population of 50 000 or less, organised into an administrative region called ‘*eup*’, which has its self-government function. Small towns that do not have an administrative organisation like a village in the west are organised into an administrative region called ‘*myeon*’.

The survey categorised participants' occupations into managers, office, service, agricultural, technical workers, and unemployed. Here we defined white-collar workers as those who hold positions as managers, professionals, or office employees. The opposite of white-collar was defined as having a blue-collar job or no job. Health screening refers to health examinations that include self-funded tests, workplace assessments, or free national health examinations. Poor access to nutrition was determined by a food survey questionnaire and defined as the percentage of people who had insufficient food due to economic problems in the past year. Limited access to hospitals means that individuals have not been able to receive examinations or treatment, excluding dental care, for various reasons in the past year.

### Statistical analysis

We presented the participants’ demographic characteristics as means (x̄) and standard deviations (SDs) or medians (MDs) and interquartile ranges (IQRs) for continuous variables and percentages and standard errors (SEs) for categorical variables, testing the normality of the data distribution using both graphical and statistical methods. We compared these characteristics across survey periods using analysis of variance (ANOVA) or the Kruskal-Wallis test for continuous variables and the χ^2^ test (χ^2^) test or χ^2^ linear trend test for categorical variables. Trends in risk factors were examined by estimating the *P*-value for the interaction term of the SES indicator and the variables identifying the four years of data in the model. The CCVD risk factors and the relationships between factors are presented using odds ratios (ORs) and 95% confidence intervals (CIs) calculated from binary logistic regression analysis adjusted for age, sex, urbanity, occupation, and obesity. Multivariable analysis was performed by including all factors that could affect CCVD. *P*-values <0.05 indicated statistical significance. All analyses were performed using R, version 3.6.3 (R Core Team, Vienna, Austria) and SPSS, version 25.0 (SPSS-PC, Chicago, IL, USA).

## RESULTS

### Trends in risk factors and CCVD prevalence from 2007 to 2018

**Figure 1:**
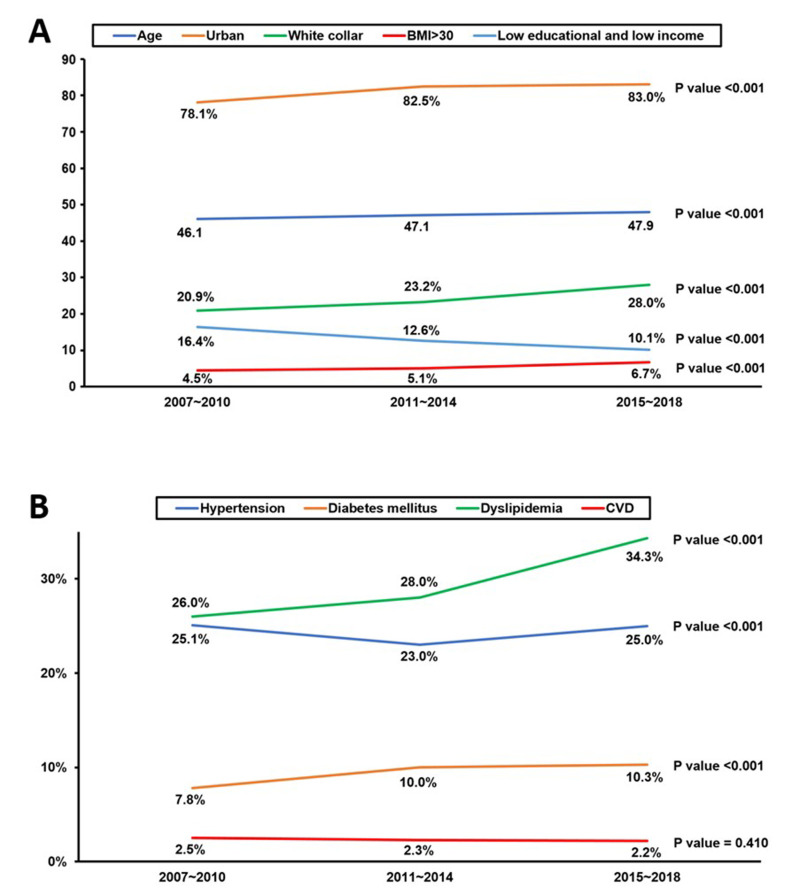
Twelve-year trends of study population characteristics and disease prevalence. **Panel A.** Twelve-year trends in age, urbanisation, white-collar employment, BMI>30, and low education and income populations. **Panel B.** Twelve-year trends of study population in hypertension, diabetes mellitus, dyslipidaemia, and CCVD. CCVD – cardio-cerebrovascular disease.

We observed a significant increase in the average age of the population from 46.1 years in 2007–20 to 47.9 years in 2015–18 (*P* < 0.001) and in urban residences from 78.1% to 83.0% (*P* < 0.001). There was a notable increase in white-collar employment from 20.9% to 28.0% (*P* < 0.001) and in participation in health screening from 54.5% to 64.7% (*P* < 0.001). Simultaneously, obesity rates increased from 4.5% to 6.7% (*P* < 0.001), while poor access to nutrition and low levels of aerobic exercise decreased significantly (*P* < 0.001). There was a significant decrease in the percentage of individuals with low education and income (group D) from 16.4% to 10.1% (*P* < 0.001) during the study period (**Figure 1**, Panel A; Table S2 in the **Online Supplementary Document**).

While the prevalence of hypertension showed only moderate changes, the prevalence of diabetes mellitus and dyslipidaemia increased significantly (*P* < 0.001). Treatment rates for hypertension and dyslipidaemia also increased significantly (*P* < 0.001). Despite these shifts, the prevalence of total CCVD, including myocardial infarction, angina, and stroke, remained unchanged over the 12 years (*P* = 0.410). The treatment rate for CCVD showed a nonsignificant increasing trend (*P* = 0.098) (**Figure 1**, Panel B; Table S2 in the **Online Supplementary Document**).

### Baseline characteristics according to education and income level

**Figure 2:**
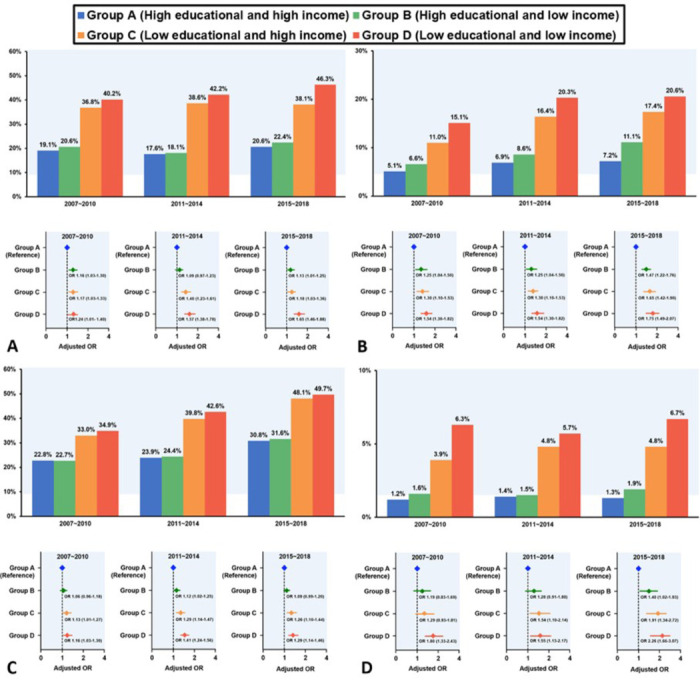
Twelve-year trends in odds ratios for disease prevalence. **Panel A.** Twelve-year comparisons of hypertension prevalence across groups A–D. **Panel B**. Twelve-year comparisons of diabetes mellitus prevalence across groups A–D. **Panel C.** Twelve-year comparisons of dyslipidaemia prevalence across groups A–D. **Panel D.** Twelve-year comparisons of CCVD prevalence across groups A–D. CCVD – cardio-cerebrovascular diseases, group A – high education and high-income group, group D – low education and low-income group.

The study population had a median age of 47.0 years (IQR = 39.0–55.0) (**Table 1**). Among the groups, individuals in the low-education and low-income group (group D) had the highest average age (MD = 56.0, IQR = 52.0–61.0), while those in the high-education and low-income group (group B) had the lowest average age (MD = 43.0, IQR = 36.0–52.0) (*P* < 0.001). Regarding gender distribution, the high-education and high-income group (group A) had the highest proportion of male participants (47.2%), while the low-education and low-income group (group D) had the lowest (33.4%) (*P* < 0.001). A similar pattern was observed for the proportion of individuals living in urban areas, those with white-collar jobs, and those currently consuming alcohol. The percentage of health screening was highest in group C and lowest in group B. Conversely, current smoking was highest in group B and lowest in group C. The proportions of those with poor access to nutrition, limited access to hospitals, poor aerobic exercise, and BMI>30 were highest in group D and lowest in group A. The prevalence of hypertension, diabetes mellitus, and dyslipidaemia, which are traditional triggers of CCVD, was consistently higher in groups C and D than in group A over 12 years **(****Figure 2**, Panels A–C**)**. Treatment rates for hypertension, diabetes mellitus, and dyslipidaemia also showed similar prevalence trends. They were highest in group D and lowest in group B (**Table 1**). The prevalence of CCVD was also higher in groups C and D compared with group A (*P* < 0.001) (**Figure 2**, panel D).

**Table 1:** Baseline characteristics according to education and income*

	Total population, (n = 47 735)	Group A, (n = 26 032)	Group B, (n = 10 502)	Group C, (n = 4955)	Group D, (n = 6246)	*P-*value
**Demographics**						
Age in years, MD (IQR)	47.0 (39.0–55.0)	43.0 (37.0–51.0)	43.0 (36.0–52.0)	56.0 (51.0–60.0)	58.0 (52.0–61.0)	<0.001
Gender, male	20 881 (43.7)	12 282 (47.2)	4737 (45.1)	1777 (35.9)	2085 (33.4)	<0.001
Urban	38 743 (81.2)	22 457 (86.3)	8522 (81.1)	3513 (70.9)	4251 (68.1)	<0.001
Job, white-collar	11 468 (24.0)	9324 (35.8)	1888 (18.0)	154 (3.1)	102 (1.6)	<0.001
Percentage of health screening	27 376 (57.3)	15 567 (59.8)	4671 (44.5)	3340 (67.4)	3798 (60.8)	<0.001
Poor access to nutrition	21 728 (45.5)	10 031 (38.5)	5638 (53.7)	2275 (45.9)	3784 (60.6)	<0.001
Limited access to hospitals	4092 (8.6)	1887 (7.2)	945 (9.0)	485 (9.8)	775 (12.4)	<0.001
Regular aerobic exercise	10 906 (22.8)	6328 (24.3)	2239 (21.3)	1128 (22.8)	1211 (19.4)	<0.001
BMI≥30	2581 (5.4)	1151 (4.4)	682 (6.5)	298 (6.0)	450 (7.2)	<0.001
Waist circumference in cm, x̄ (SD)	81.7 (9.5)	80.9 (9.4)	81.5 (9.7)	83.3 (8.8)	83.9 (9.4)	<0.001
Current drinking	33 661 (70.5)	19 895 (76.4)	7326 (69.8)	2980 (60.1)	3460 (55.4)	<0.001
Current smoking	11 824 (24.8)	6313 (24.3)	2822 (26.9)	1103 (22.3)	1586 (25.4)	<0.001
**Cardiovascular comorbidity**						
Hypertension	11 636 (24.4)	4983 (19.1)	2137 (20.3)	1868 (37.7)	2648 (42.4)	<0.001
*Treatment*	5954 (51.2)	2186 (43.9)	927 (43.4)	1125 (60.2)	1716 (64.8)	<0.001
*Above 140/90 mm Hg*	7188 (15.1)	3287 (12.6)	1414 (13.5)	1049 (21.2)	1438 (23.0)	<0.001
Diabetes mellitus	4446 (9.3)	1670 (6.4)	929 (8.8)	713 (14.4)	1134 (18.2)	<0.001
*HbA1c in %*, *MD (IQR)*	6.0 (5.0–6.0)	6.0 (5.0–6.0)	6.0 (5.0–6.0)	6.0 (6.0–6.0)	6.0 (6.0–6.0)	<0.001
*Treatment*	2322 (52.2)	774 (46.3)	422 (45.4)	409 (57.4)	717 (63.2)	<0.001
*HbA1c >7.0%*	3256 (6.8)	1244 (4.8)	676 (6.4)	519 (10.5)	817 (13.1)	<0.001
Dyslipidemia	14 041 (29.4)	6767 (26.0)	2770 (26.4)	1936 (39.1)	2568 (41.1)	<0.001
*Total cholesterol in mg/dl*, *x̄ (SD)*	192.9 (36.1)	192.0 (35.2)	191.6 (36.2)	196.6 (37.0)	195.3 (38.3)	<0.001
*Triglyceride in mg/dl*, *MD (IQR)*	110.0 (74.0–167.0)	105.0 (71.0–161.0)	108.0 (73.0–168.0)	118.0 (80.0–176.0)	123.0 (83.0–182.0)	<0.001
*HDL cholesterol in mg/dl, x̄ (SD)*	50.2 (12.1)	50.7 (12.2)	50.2 (12.2)	49.2 (11.9)	48.9 (12.0)	<0.001
*LDL cholesterol in mg/dl, x̄ (SD)*	106.3 (45.1)	105.7 (44.7)	100.1 (49.1)	114.8 (39.4)	112.4 (42.1)	<0.001
*Treatment*	2787 (19.8)	1096 (16.2)	438 (15.8)	525 (27.1)	728 (28.3)	<0.001
*LDL>130mg/dl*	14 119 (29.6)	7444 (28.6)	2780 (26.5)	1757 (35.5)	2138 (34.2)	<0.001
MI or angina	643 (1.3)	210 (0.8)	93 (0.9)	130 (2.6)	210 (3.4)	<0.001
*Treatment*	492 (76.5)	151 (71.9)	68 (73.1)	102 (78.5)	171 (81.4)	0.093
Stroke	522 (1.1)	131 (0.5)	92 (0.9)	96 (1.9)	202 (3.2)	<0.001
*Treatment*	330 (63.2)	79 (60.3)	59 (64.1)	58 (60.4)	134 (66.3)	0.679
CCVD (MI, angina, or stroke)	1113 (2.3)	330 (1.3)	177 (1.7)	218 (4.4)	388 (6.2)	<0.001
*Treatment*	794 (71.3)	225 (68.2)	125 (70.6)	155 (71.1)	289 (74.5)	0.016

BMI – body mass index, CCVD – cardio-cerebrovascular diseases, DBP – diastolic blood pressure, HDL – high-density lipoprotein, IQR – interquartile range, LDL – low-density lipoprotein, MD – median, MI – myocardial infarction, Regular aerobic exercise – 30 min 5 d a week, SBP – systolic blood pressure, SD – standard deviation, x̄ – mean

*Presented as n (%) unless specified otherwise.

### Risk factors associated with CCVD

For the total population (n = 47 735), low education and low income were significant risk factors for CCVD, with ORs of 4.02 (95% CI = 3.59–4.52) and 1.98 (95% CI = 1.77–2.23), respectively, in the univariate analysis (**Table 2**). In the multivariable analysis, after adjusting for all other available factors influencing CCVD prevalence, low education remained significantly associated with CCVD prevalence (OR = 1.24; 95% CI = 1.04–1.47, *P* < 0.001), as did low income (OR = 1.14; 95% CI = 1.02–1.28, *P* = 0.017). Age, being male, blue-collar or no job, poor access to nutrition, current alcohol use, current smoking, hypertension, diabetes mellitus, and dyslipidaemia continued to show significant associations with CCVD.

**Table 2:** Risk factors associated with CCVD in the sample (n = 47 735)

	Univariate analysis		Multivariable analysis	
**Variables**	**OR (95% CI)**	***P-*value**	**OR (95% CI)**	***P*-value**
Low education	4.02 (3.59–4.52)	<0.001	1.24 (1.04–1.47)	<0.001
Low income	1.98 (1.77–2.23)	<0.001	1.14 (1.02–1.28)	0.017
Age	1.48 (1.28–1.65)	<0.001	1.10 (1.08–1.11)	<0.001
Gender, male	1.62 (1.44–1.83)	<0.001	1.95 (1.64–2.32)	<0.001
Rural area	1.38 (0.93–1.22)	<0.001	1.02 (0.93–1.19)	0.388
Job, blue-collar/no job	2.02 (1.70–2.42)	<0.001	1.10 (1.05–1.15)	<0.001
No health screening	1.45 (1.28–1.65)	<0.001	1.00 (0.88–1.13)	0.386
Poor access to nutrition	1.36 (1.25–1.48)	<0.001	1.19 (1.05–1.34)	0.006
Limited access to hospitals	1.16 (0.95–1.41)	0.135	1.22 (0.98–1.51)	0.068
Poor aerobic exercise*	1.17 (0.95–1.33)	0.117	1.01 (0.87–1.16)	0.340
BMI>30	1.54 (1.29–1.98)	<0.001	1.29 (0.98–1.68)	0.063
Current alcohol use	1.31 (1.14–1.45)	<0.001	1.14 (1.01–1.30)	0.042
Current smoking	1.35 (1.18–1.53)	<0.001	1.21 (1.05–1.43)	0.010
Hypertension	4.62 (4.11–5.23)	<0.001	1.89 (1.61–2.22)	<0.001
Diabetes mellitus	4.32 (3.77–4.92)	<0.001	1.74 (1.46–2.06)	<0.001
Dyslipidaemia	2.59 (2.30–2.92)	<0.001	1.52 (1.30–1.77)	<0.001

BMI – body mass index, CI – confidence interval, CCVD – cardio-cerebrovascular diseases, OR – odds ratio

*Regular aerobic exercise was defined as less than 30 min, five days a week.

### Relationship between risk factors and CCVD by SES

**Figure 3:**
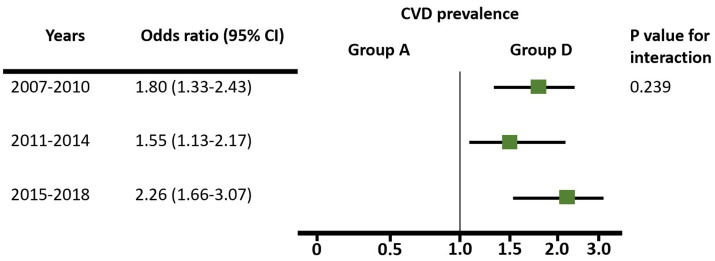
Twelve-year trends in odds ratios for CCVD prevalence between groups A and D. Group A – high educational and high-income group, group D – low education and low-income group.

Analysing the adjusted OR of age, sex, urban living, job, and obesity, when comparing group A as the reference, we confirmed that the prevalence of hypertension, diabetes mellitus, and dyslipidaemia was gradually higher (Table S3 in the **Online Supplementary Document**). Notably, compared to group A, groups C and D showed a significantly higher disease prevalence with hypertension, diabetes mellitus, and dyslipidaemia, adjusting for several factors, with no change in this trend over 12 years (**Figure 2**, Panels A–C). Regarding myocardial infarction, angina, and stroke, which were CCVD components, the ORs of groups C and D were significantly higher than that of group A, with the trend remaining unchanged through 12 years (**Figure 2**, panel D). Group D showed higher disease prevalence compared to group A, with an OR of 1.53 (95% CI = 1.22–1.91) for myocardial infarction or angina, 2.54 (95% CI = 1.97–3.28) for stroke, and 1.92 (95% CI = 1.61–2.28) for CCVD. Regarding CCVD, when comparing groups D and A, the OR was 1.80 (95% CI = 1.33–2.43) from 2007 to 2010, 1.55 (95% CI = 1.13–2.17) from 2011 to 2014, and 2.26 (95% CI = 1.66–3.07) from 2015 to 2018. The *P*-value for interaction was 0.239, suggesting that the difference in CCVD was consistently higher in group D without statistically significant changes over 12 years (**Figure 3**).

## DISCUSSION

In this cross-sectional study, we used data from the KNHANES to examine the prevalence of CCVD and its association with multiple risk factors, categorising individuals into four groups based on education and income levels over 12 years. Our main findings show that, despite Korea’s transition to an ageing, urbanised society with an increase in white-collar occupations and obesity, and a decrease in the proportion of the population with low education and low income from 2007 to 2018, the overall prevalence of CCVD among economically active individuals remained stable. Furthermore, we identified both low education and low income as significant determinants of CCVD prevalence, in addition to traditional risk factors such as age, sex, hypertension, diabetes mellitus, and dyslipidaemia. Compared with the high education and high-income group, the lowest SES group, which is characterised by both low education and low income, had a higher prevalence of CCVD, with no clear change in trends over the 12-year study period.

### Complexity of socioeconomic disparities

CCVDs, including ischaemic heart disease and stroke, remain the leading cause of death worldwide [12], with SES, a composite indicator that includes income, education, race, and occupation, significantly influencing its prevalence [4,5]. SES disparities in CCVD or related risk factors leading to higher mortality have been well documented in high-income Western countries. Korea, like Taiwan, mitigates these effects through universal health insurance coverage, reducing the impact of SES on disease prevalence compared to Western countries [13]. With the cross-sectional analysis, we sought to understand the relationship between SES disparities and CCVD prevalence, including risk factors, in the Korean population over 12 years. Korea has experienced a gradual upward trend in ageing, urbanisation, white-collar employment, obesity, diabetes mellitus, and dyslipidaemia, similar to trends in other high-income countries, while the proportion of the population with low education and income levels has decreased [14]. Despite the ageing population, urbanisation, and increased prevalence of obesity, diabetes mellitus, and dyslipidaemia, our analysis did not show a significant change in CCVD prevalence. This stability may be attributed to improved access to health care and interventions with better medications and lifestyle changes between 2007 and 2018. Specifically, we analysed that the increased use of health screening, aerobic exercise, and improved treatment of hypertension and dyslipidaemia, combined with reductions in smoking rates and improvements in dietary habits, may play a key role in mitigating the increase in CCVD prevalence expected from our data. In addition, the introduction of national health policies focussing on preventive care and regular monitoring of high-risk groups may have contributed to this stabilisation by enabling early detection and management of CCVD-related conditions in Korea [15]. The socioeconomic dynamics in Korea, characterised by a robust public health system and comprehensive health insurance, provide a unique context that helps buffer the adverse effects of SES disparities on health outcomes [16]. However, the complexity of these socioeconomic disparities requires further investigation to understand the interplay of detection, persistence, and resolution of health inequalities over time. This multifaceted approach is crucial in addressing the long-term impacts of SES on CCVD prevalence.

### Persistent health disparities

Although the overall prevalence of CCVD did not change over the 12 years, the disparity in CCVD prevalence between SES groups remained constant, with the lowest education and income group (group D) consistently having the highest prevalence compared with the highest education and income group (group A). Group D, which is characterised by being older, having a higher proportion of women, living in rural areas, having less white-collar employment, being less physically active, having a higher BMI, not adhering to a healthy diet, and having the fewest hospital visits in the past year, also had significantly higher rates of hypertension, diabetes mellitus, and dyslipidaemia. However, it also had higher treatment rates for these conditions. Nevertheless, the treatment rate for dyslipidaemia was low across the board, at only 28% in group D. Thus, improving the management of dyslipidaemia is another critical option for reducing future CCVD prevalence. Furthermore, the interplay of various social and economic factors, including limited access to preventive health care services, financial constraints, and lower health literacy, worsens these disparities [17,18]. Addressing these issues requires targeted public health interventions that focus on improving health care access, providing financial assistance, and enhancing health education among low SES populations [19,20].

### Supporting evidence

Our findings are consistent with previous research highlighting the association between lower SES and increased CCVD and its risk factors [21]. Individuals with lower SES often face barriers to accessing quality health care, resulting in delayed or inadequate care and contributing to a higher prevalence of CCVD in these communities. Education and income levels play a crucial role in lifestyle choices that affect CCVD risk, including dietary habits, physical activity, smoking, and alcohol consumption [22]. Populations with lower levels of education and income often engage in unhealthy lifestyle behaviours, such as poor dietary choices, limited physical activity, and higher rates of smoking and excessive alcohol consumption, all of which are established risk factors for CCVD [23]. Modifiable lifestyle factors such as diet and physical activity are expected to play a central role in reducing health inequalities, particularly among lower-income and education groups [24]. However, the challenge of balancing the cost of living with the cost of healthier food options can prevent individuals and families with lower incomes from meeting dietary guidelines. This situation underscores the importance of government financial support to facilitate access to healthy diets and thereby promote cardiovascular health in these vulnerable groups, where the need for intervention is both critical and impactful. Additionally, social determinants such as housing stability, employment status, and access to community resources also play significant roles in influencing health outcomes and should be considered in public health strategies [19].

### Limitations

This study has several limitations. First, it had a cross-sectional design, which made it difficult to determine the exact causal relationships between SES and CCVD risk factors. We also used data from a sample survey, not a complete survey. Therefore, it is unknown whether there are overlapping populations in our data, and causality should be used in interpreting the result for the target population. Second, KNHANES is a self-report survey; therefore, there may be an assessment bias. In particular, the treatment rate for dyslipidaemia was very low compared with the treatment rate for CCVD. Therefore, there is a high probability that patients are unaware that dyslipidaemia medications are included in treatment. Third, the generalisability of our results to the entire Korean population is limited because our study included only individuals aged 30–64, and institutionalised older adults were excluded. Another limitation of our study is potential survivor bias. Our analysis did not include individuals who experienced sudden death or those who did not survive to reach the age criteria for inclusion. This could lead to an underestimation of the true burden of CCVD in the population, as those with more severe diseases who may have died earlier are not represented. As our study is a cross-sectional study investigating prevalence, the lack of mortality is a critical issue. Future research should consider including mortality data to provide a more accurate picture of CCVD prevalence and its association with socioeconomic factors. Finally, we could not examine trends in SES disparities in CCVD risk factors before 2007 because the KNHANES survey data from this period did not include information on laboratory tests.

## CONCLUSIONS

In this cross-sectional study based on data from Korea, one of emerging high-income countries, the prevalence of CCVD remained unchanged from 2007 to 2018, despite the ageing of its population and increasing trends in obesity. Individuals with both low education and income had consistently higher CCVD rates compared to higher education and income populations, indicating that disparities have not changed over 12 years. There is a need to improve access to medical care for low-education and income groups to eliminate these disparities, with a focus on controlling hypertension, diabetes, and hyperlipidemia, and promoting obesity management through support for healthier diets.

## Additional material


Online Supplementary Document
