# Supplementary material for: Socioeconomic disparities and cardio-cerebrovascular diseases: A nationwide cross-sectional study
Source: J Glob Health. 2024 Oct 11;14:04210. doi: 10.7189/jogh.14.04210 (PMC11466497; doi:10.7189/jogh.14.04210)

Supplementary Material

Socioeconomic disparities and cardio-cerebrovascular disease: A nationwide population-based cross-sectional study

Ji Woong Roh, MD, PhD[1†]; SungA Bae, MD, PhD[1†]; Moon-Hyun Kim, MD[1]; Je-Wook Park, MD[1]; Seok-Jae Heo, PhD[2]; Minkwan Kim, MD, PhD[1]; Oh-Hyun Lee, MD[1]; Yongcheol Kim, MD, PhD[1]; Eui Im, MD[1]; Jae-Sun Uhm, MD, PhD[2]; Jung In Hyun, MD, PhD[1];

Deok-Kyu Cho, MD[1*]; Donghoon Choi, MD, PhD[3*]

CONTENTS

Supplementary Table S1. The monthly income quartile of the sample population by year.

Supplementary Table S2. Trends in prevalence between 2007 and 2018

Supplementary Table S3. Relationship between cardiovascular risk factors and CVD by education and income

Supplementary Figure S1. Study flow chart

Supplementary Figure S2. Difference in CVD prevalence by education level (A) income level (B) by 4 division

Supplementary Table S1. The monthly income quartile of the sample population by year.

	Quartile (2007-2009)				Quartile (2010-2012)				Quartile (2013-2015)				Quartile (2016-2018)			
	1	2	3	4	1	2	3	4	1	2	3	4	1	2	3	4
Men																
30-34	664.8	943.6	1218.2	1728.3	918.6	1236.7	1478.7	2172.9	1001.3	1361.8	1848	2480.7	1295.5	1732.4	2287.3	3058.2
35-39	712.5	985.3	1274.9	1732.2	939.9	1292.7	1500.3	2180.4	1030.8	1399.5	1788.9	2412	1257.6	1667.8	2150.5	2850.5
40-44	673.9	1045.4	1354.5	1837.7	979.2	1327.1	1572.4	2435.8	1014.9	1442.8	1940	2559.8	1238.7	1737	2244.6	2948.1
45-49	626.8	949.2	1274.1	1912.2	892.9	1316.8	1585.3	2312.2	935	1440.6	1900.9	2680.8	1235.7	1743.5	2326.3	3103.9
50-54	554	917.1	1349.4	1900.4	876.6	1390.5	1721.5	2816.9	868.9	1359.5	1944.6	2822.1	1178.5	1858.3	2544.9	3578.7
55-59	456.3	796.8	1169.7	1807.7	875.4	1347.8	1629.6	2683.3	785.2	1246.1	1841.1	2705.2	1071.3	1794.1	2445.6	3437.5
60-64	379.8	607.8	875.6	1336.7	658.9	1059.1	1308.5	2209.4	618.7	1066.1	1502.9	2281.3	854.5	1336.9	1895.1	2741.7
Women																
30-34	697.6	933.9	1201.1	1598.3	935.9	1247.1	1577.2	2161.3	973.8	1358.7	1787.8	2521.5	1211.2	1611.8	2071.2	2857.2
35-39	683	997.8	1312.3	1780.6	951.4	1290.9	1623.9	2248.1	935.2	1351.6	1733.3	2308.2	1195.3	1611.4	2072.8	2766.5
40-44	669.7	985.1	1315.6	1905.6	859.2	1260.9	1703.7	2478.6	1009.2	1459.1	1962.5	2676.3	1245.8	1791.8	2316.7	3038.9
45-49	556.7	896.5	1242.3	1894.6	894.1	1374.6	1860.6	2619.8	851.6	1303.3	1864.6	2727.7	1151.9	1730.2	2373.1	3154.5
50-54	496.9	815.1	1205.1	1849.8	878.8	1329.2	1828.3	2701.4	856.2	1367.3	1957.6	2914.3	1158.9	1835.1	2448.6	3429.9
55-59	401.1	643.9	951.5	1466.9	699.9	1102.8	1580.2	2325.7	696.8	1119.9	1611.2	2436.5	940.9	1488.2	2123.3	2959.2
60-64	279.5	463.7	681	1160.3	461.3	753.4	1142.3	1728.4	494.5	821.9	1236.5	1829.5	687.2	1151.6	1643	2414.2

Unit: Dollars

Supplementary Table S2. Trends of prevalence between 2007 and 2018

	Total population (N =47,735)	2007~2010 (N =16,246)	2011~2014 (N =15,768)	2015~2018 (N =15,721)	*P* value
Demographics					
Age (years)	47.0 (39.0-55.0)	46.0 (38.0-54.0)	47.0 (39.0-55.2)	48.0 (40.0-56.0)	<0.001
Gender, male	20881 (43.7%)	7152 (44.0%)	6819 (43.2%)	6910 (44.0%)	0.303
Urban	38743 (81.2%)	12690 (78.1%)	13001 (82.5%)	13052 (83.0%)	<0.001
Job, white-collar	11468 (24.0%)	3402 (20.9%)	3666 (23.2%)	4400 (28.0%)	<0.001
Percentage of health screening	27376 (57.3%)	8856 (54.5%)	8348 (52.9%)	10172 (64.7%)	<0.001
Poor access to nutrition	21728 (45.5%)	8029 (49.4%)	7563 (48.0%)	6136 (39.0%)	<0.001
Limited access to hospitals	4092 (8.6%)	767 (4.7%)	2018 (12.8%)	1307 (8.3%)	<0.001
*Regular aerobic exercise	10906 (22.8%)	2097 (12.9%)	2467 (15.6%)	6342 (40.3%)	<0.001
BMI > 30	2581 (5.4%)	723 (4.5%)	802 (5.1%)	1056 (6.7%)	<0.001
Current drinking	33661 (70.5%)	11424 (70.3%)	11034 (70.0%)	11203 (71.3%)	<0.001
Current smoking	11824 (24.8%)	5591 (34.4%)	3142 (19.9%)	3091 (19.7%)	<0.001
Low educational and low income	6,246 (13.1%)	2,666 (16.4%)	1,992 (12.6%)	1,588 (10.1%)	<0.001
Cardiovascular comorbidity					
Hypertension	11636 (24.4%)	4083 (25.1%)	3622 (23.0%)	3931 (25.0%)	<0.001
Treatment	5954 (51.2%)	1940 (47.5%)	1844 (50.9%)	2170 (55.2%)	<0.001
Above >140/90mmHg	7188 (15.1%)	2755 (17.0%)	2244 (14.2%)	2189 (13.9%)	<0.001
Diabetes mellitus	4446 (9.3%)	1265 (7.8%)	1569 (10.0%)	1612 (10.3%)	<0.001
Treatment	2322 (52.2%)	720 (56.9%)	749 (47.7%)	853 (53.0%)	<0.001

	Total population (N =47,735)	2007~2010 (N =16,246)	2011~2014 (N =15,768)	2015~2018 (N =15,721)	*P* value
HbA1c > 7.0%	3256 (6.8%)	854 (5.3%)	1234 (7.8%)	1168 (7.4%)	<0.001
Dyslipidemia	14041 (29.4%)	4232 (26.0%)	4414 (28.0%)	5395 (34.3%)	<0.001
Treatment	2787 (19.8%)	522 (12.3%)	845 (19.1%)	1420 (26.3%)	<0.001
LDL > 130mg/dL	14119 (29.6%)	4446 (27.4%)	4411 (28.0%)	5262 (33.5%)	<0.001
MI or angina	643 (1.3%)	245 (1.5%)	205 (1.3%)	193 (1.2%)	0.077
Treatment	492 (76.5%)	176 (71.8%)	163 (79.5%)	153 (79.3%)	0.090
Stroke	522 (1.1%)	169 (1.0%)	172 (1.1%)	181 (1.2%)	0.634
Treatment	330 (63.2%)	104 (61.5%)	105 (61.0%)	121 (66.9%)	0.454
CVD (MI, angina or stroke)	1113 (2.3%)	399 (2.5%)	362 (2.3%)	352 (2.2%)	0.410
Treatment	794 (71.3%)	272 (68.2%)	258 (71.3%)	264 (75.0%)	0.098

*5 days/week, daily 30 minutes

Values are presented as mean ± SD or n (%).

BMI, body mass index; CVD, cardio-cerebrovascular disease; DBP, diastolic blood pressure; HDL, high density lipoprotein; LDL, low density lipoprotein; MI, myocardial infarction; SBP, systolic blood pressure.

Supplementary Table S3. Relationship between cardiovascular risk factors and CVD by education and income

	Group A (N = 10,502)	Group B (N = 10,502)	Group C (N = 4,955)	Group D (N = 6,246)
		*Adjusted OR (95% CI)	*Adjusted OR (95% CI)	*Adjusted OR (95% CI)
Hypertension	Reference	1.13 (1.05-1.20)	1.28 (1.19-1.39)	1.49 (1.39-1.61)
Diabetes mellitus	Reference	1.28 (1.15-1.43)	1.43 (1.30-1.57)	1.56 (1.42-1.73)
Dyslipidemia	Reference	1.10 (1.04-1.16)	1.15 (1.07-1.23)	1.20 (1.12-1.28)
MI or angina	Reference	1.06 (0.82-1.37)	1.36 (1.06-1.73)	1.53 (1.22-1.91)
Stroke	Reference	1.64 (1.24-2.17)	1.88 (1.40-2.51)	2.54 (1.97-3.28)
CVD (MI, angina or stroke)	Reference	1.29 (1.07-1.57)	1.58 (1.30-1.92)	1.92 (1.61-2.28)

*Adjusted: age, sex, urban, job, obesity

CVD, cardio-cerebrovascular disease; MI, myocardial infarction.

Supplementary Figure S1. Study flow chart

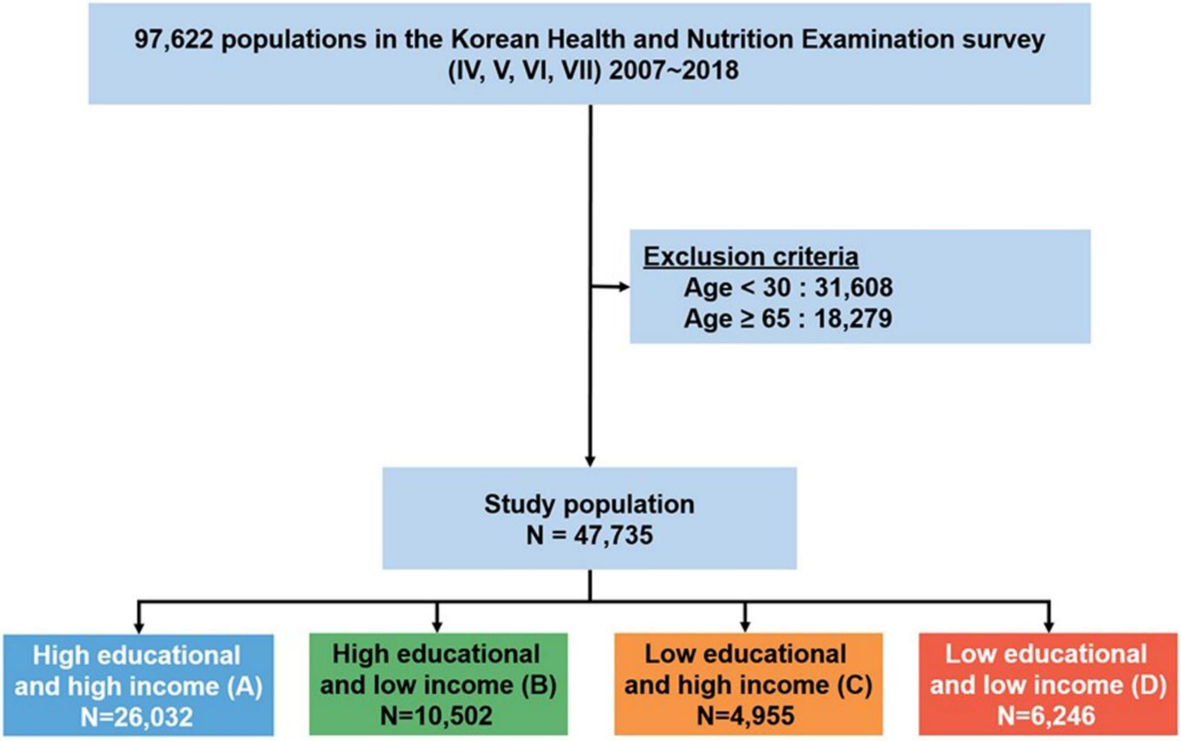

Supplementary Figure S2. Difference in CVD prevalence by education level (A) income level (B) by 4 division

(A)

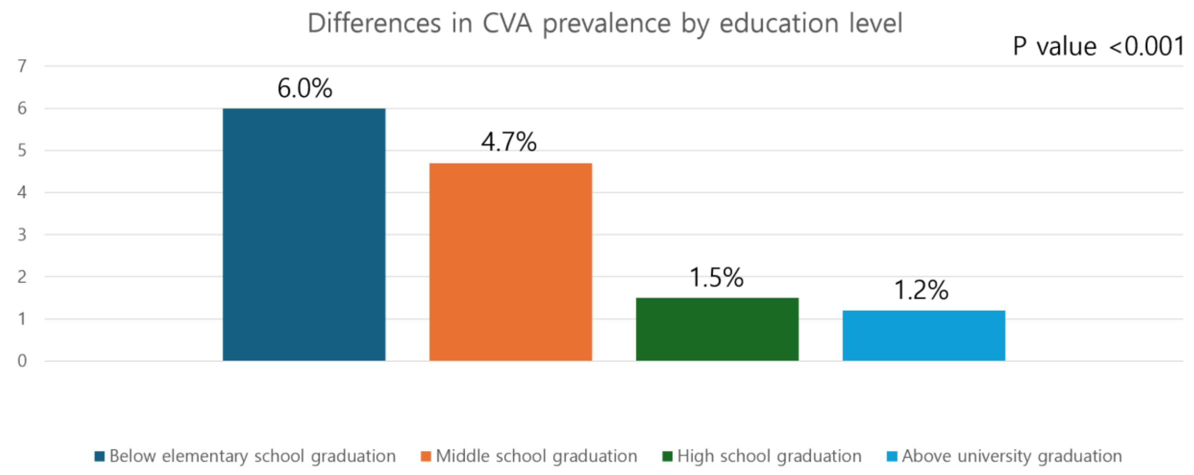

(B)

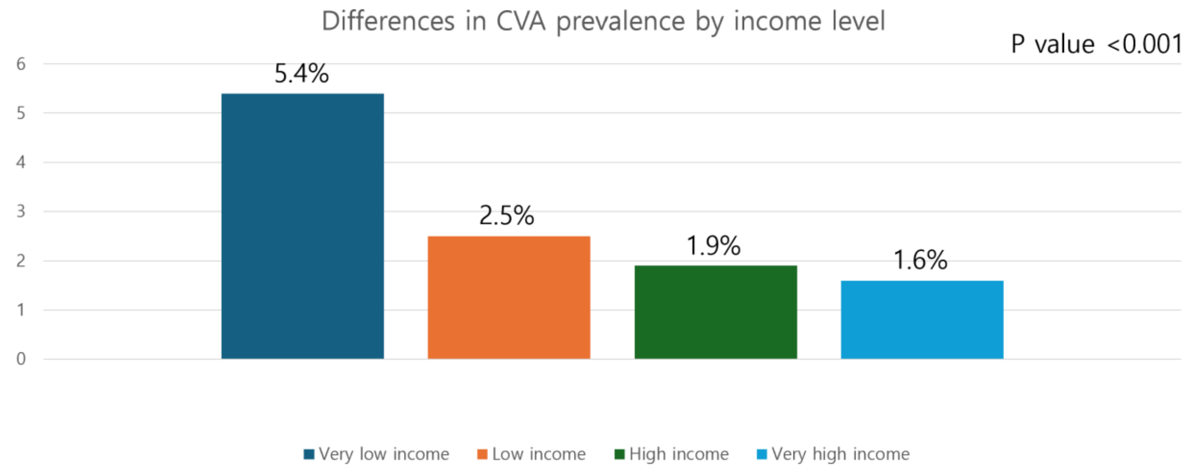

Supplement: Online Supplementary Document [file jogh-14-04210-s001.pdf]
